# Neonatal reference intervals for thyroid stimulating hormone and free thyroxine assayed on a Siemens Atellica® IM analyzer: a cross sectional study

**DOI:** 10.1186/s12902-023-01367-6

**Published:** 2023-05-19

**Authors:** Geoffrey Omuse, David Kawalya, Patrick Mugaine, Assumpta Chege, Daniel Maina

**Affiliations:** Department of Pathology, Aga Khan University Hospital Nairobi, Nairobi, Kenya

**Keywords:** Neonatal reference intervals, TSH, FT4, Congenital hypothyroidism, Free thyroxine, Thyroid stimulating hormone

## Abstract

**Background:**

Deriving population specific reference intervals (RIs) or at the very least verifying any RI before adoption is good laboratory practice. Siemens has provided RIs for thyroid stimulating hormone (TSH) and free thyroxine (FT4) determined on their Atellica® IM analyzer for all age groups except the neonatal age group which provides a challenge for laboratories that intend to use it to screen for congenital hypothyroidism (CH) and other thyroid disorders in neonates. We set out to determine RIs for TSH and FT4 using data obtained from neonates undergoing routine screening for CH at the Aga Khan University Hospital, Nairobi, Kenya.

**Methodology:**

TSH and FT4 data for neonates aged 30 days and below were extracted from the hospital management information system for the period March 2020 to June 2021. A single episode of testing for the same neonate was included provided both TSH and FT4 were done on the same sample. RI determination was performed using a non-parametric approach.

**Results:**

A total of 1243 testing episodes from 1218 neonates had both TSH and FT4 results. A single set of test results from each neonate was used to derive RIs. Both TSH and FT4 declined with increase in age with a more marked decline seen in the first 7 days of life. There was a positive correlation between logFT4 and logTSH (r_s_ (1216) = 0.189, p = < 0.001). We derived TSH RIs for the age groups 2–4 days (0.403–7.942 µIU/mL) and 5–7 days (0.418–6.319 µIU/mL), and sex specific RIs for males (0.609–7.557 µIU/mL) and females (0.420–6.189 µIU/mL) aged 8–30 days. For FT4, separate RIs were derived for the age groups 2–4 days (1.19–2.59 ng/dL), 5–7 days (1.21–2.29 ng/dL) and 8–30 days (1.02–2.01 ng/dL).

**Conclusion:**

Our neonatal RIs for TSH and FT4 are different from those published or recommended by Siemens. The RIs will serve as a guide for the interpretation of thyroid function tests in neonates from sub-Saharan Africa where routine screening for congenital hypothyroidism using serum samples is done on the Siemens Atellica® IM analyzer.

## Background

Congenital hypothyroidism (CH) is a known cause of intellectual disability that can be prevented if diagnosed and treated early. For this reason, testing for CH has been incorporated as part of newborn screening programs. Unfortunately, most babies worldwide do not have access to a newborn screening program meaning that most cases of CH go undiagnosed [1]. This is particularly so in sub-Saharan Africa (SSA) where newborn screening programmes are not established hence the true burden of CH is unknown. In order to screen for hypothyroidism, three approaches are used. The first two involve measuring either thyroid stimulating hormone (TSH) or thyroxine (T4) with reflex testing of the other when the result is abnormal. The ideal approach however would be simultaneous detection of both elevated TSH and low serum free thyroxine (FT4) in order to overcome the limitations of testing either one alone [2].

At the Aga Khan University Hospital Nairobi (AKUHN) our screening approach involves measurement of TSH with or without FT4 [2]. Correct interpretation of these tests is dependent upon use of appropriate reference intervals (RIs). Racial differences in TSH and T4 levels have been described with blacks in the US found to have lower levels compared to whites and Mexican Americans highlighting the need for population specific RIs [3]. We have previously demonstrated that neonatal RIs in Kenya for TSH and FT4 determined using a Roche assay differ from those recommended by Roche which were derived from neonates in Germany [4]. Specifically, the TSH and FT4 upper limits for the Kenyan neonates were lower meaning that use of the Roche RIs would have misclassified some babies as being normal denying them an opportunity for subsequent follow up to confirm whether or not they had CH.

Deriving population specific RIs or at the very least verifying any RI before adoption is good laboratory practice [5]. This becomes even more important when setting up a CH screening program where it is vital that cut-offs for TSH and FT4 that are used are optimized to increase the diagnostic accuracy of the screening program. Lack of standardization of thyroid function tests (TFTs) results in significant variations in test values across analyzers from different manufacturers hence patients with thyroid disorders should have TFTSs done on the same analyzer using the same test methodology especially when monitoring response to treatment. Manufacturer recommended RIs for these tests also vary with greater variation seen for FT4 compared to TSH [6].

Siemens has provided RIs for TSH and FT4 determined on their Atellica® IM analyzer for all age groups except the neonatal age group which provides a challenge for laboratories that intend to use it to screen for CH and other thyroid disorders in neonates. For children aged 1–23 months, the Atellica® IM recommended TSH and FT4 RIs are 0.87–6.15 µIU/mL and 0.94–1.44 ng/dL respectively. To the best of our knowledge the only published study that reports Atellica® IM RIs for these tests is the CALIPER study which has data from a large multi-ethnic Canadian cohort of children. They derived RIs from birth to 18 years of 0.65–4.41 µIU/mL and 1.04–1.64 ng/dL for TSH and FT4 respectively [7]. However, having a single age band for RIs from birth to 18 years is not realistic given that TSH and FT4 values decline with increase in age in the neonatal period with the most significant changes seen within the first week of life necessitating neonatal specific RIs [8].

There are ethical concerns around carrying out studies in vulnerable populations which includes children. For this reason, indirect methods of deriving paediatric RIs are a reasonable option especially for tests that are routinely done as part of wellness checks or screening for diseases with a relatively low incidence. Several studies have taken this approach when deriving RIs for TFTs in children [4, 9, 10]. We set out to determine RIs for TSH and FT4 using data obtained from neonates undergoing routine screening for CH at the Aga Khan University Hospital, Nairobi, Kenya following our transition from cobas6000® Roche to Siemens Atellica® IM analyzers.

## Methods

This was a cross sectional study using data collected between March 2020 and June 2021 at the Aga Khan University Hospital, Nairobi (AKUHN), a 300 bed tertiary hospital located in Kenya that has a network of 50 satellite clinics in Kenya, Tanzania and Uganda. The paediatric department offers routine screening for CH to all healthy babies born in the hospital and all neonates seen in the well-baby clinics with an option for opting out. Either TSH alone or in combination with FT4 is offered based on the attending doctor’s preference. The testing is carried out on venous blood collected in serum tubes that are centrifuged at 3000 g for 10 min in the main laboratory and analyzed on a real time basis. All TFTs are assayed on a Siemens Atellica® IM analyzer. The TSH assay is a third-generation assay that employs anti-fluorescein isothiocyanate (FITC) monoclonal antibody covalently bound to paramagnetic particles, an FITC-labeled anti-TSH capture mouse monoclonal antibody, and a tracer consisting of a proprietary acridinium ester and an anti‑TSH mouse monoclonal antibody conjugated to bovine serum albumin for chemiluminescent detection. The analytical measurement range of the assay is 0.008-150.000 µIU/ml. The FT4 assay is a competitive immunoassay using direct chemiluminescent technology. FT4 in the patient sample competes with acridinium-ester-labeled thyroxine in the reagent for a limited amount of biotinylated rabbit polyclonal anti-T4 antibody. Biotin-labeled anti‑T4 is bound to avidin that is covalently coupled to paramagnetic particles in the solid phase. The analytical measurement range of the assay is 0.1–12.0 ng/dL.

The AKUHN laboratory is accredited by the College of American Pathologists (CAP) and has ISO 15189:2012 accreditation from the South African National Accreditation System. All tests in the laboratory including TFTs are enrolled to an external quality assurance programme offered by CAP with excellent performance. The laboratory runs two levels of a third party Biorad Immunoassay Plus control. The cumulative coefficient of variation (CV) for FT4 was 3.2% and 3.7% at mean concentrations of 1.18 and 3.44 ng/dL respectively. For TSH, the cumulative CV was 3.6% and 3.0% at mean concentrations of 0.69 and 27.92 µIU/mL respectively. A waiver from full ethics review was granted by the Aga Khan University human research and ethics committee.

### Data analysis

The Siemens Atellica® IM analyzer TSH and FT4 data for neonates aged 30 days and below were extracted from the hospital management information system (HMIS). Date of birth as captured on the HMIS was used to determine the neonates age in days. Only the most recent TSH and FT4 results were included for neonates who had the tests done more than once provided both TSH and FT4 data were available. The neonates were categorized into 3 age groups (2–4, 5–7 and 8–30 days) based on the rapid decline in TSH and FT4 values in the first 7 days as seen on a locally weighted scatterplot smoothing (LOWESS) graph with 90% fit. Independent median test was used to compare medians for FT4 and TSH across age and sex stratifications. Age and sex specific RIs were subsequently determined based on statistically significant differences in the respective stratifications while taking into consideration the recommendation by the clinical laboratory standards institute (CLSI) of a minimum of 120 individuals per stratification [5]. RI determination was performed using a non-parametric approach that captures the mid 95% of reference values. Reference Value Advisor v2.1 (National Veterinary School, Toulouse, France) which is a set of Microsoft excel macros was used for derivation of RIs [11]. Spearman’s correlation was used to determine the correlation between logTSH and logFT4. A linear regression plot was used to graphically represent the relationship between logTSH and logFT4. This was also done for logTSH and FT4 in order to compare our data with what has been published as the relationship between logTSH and FT4. Inferential statistical analysis was performed using IBM SPSS Statistics for Windows, Version 23.0. (Armonk, NY, IBM Corp). A p-value less than 0.05 was considered statistically significant.

## Results

A total of 1243 testing episodes from 1218 neonates had both TSH and FT4 results. Twenty-five neonates had results of TSH and FT4 from 2 different sample collections in the neonatal period for which only the most recent set of results was retained leaving 1218 unique sets of data for derivation of RIs. There was no data for neonates below 2 days of age. TSH results were lower in female neonates as shown in Table 1. The distribution of TSH and FT4 was non-Gaussian.

**Table 1:** Summary of TSH and FT4 results

		Male (n = 633)	Female (n = 585)	All (N = 1218)	p-value (Male vs. Female)
**Median (IQR)**	**TSH (µIU/mL)**	2.308 (2.006)	1.976 (1.718)	2.103 (1.918)	0.000
	**FT4 (ng/dL)**	1.69 (0.43)	1.69 (0.40)	1.69 (0.41)	0.847

Key: FT4-free thyroxine, IQR-interquartile range, TSH-thyroid stimulating hormone

**Fig. 1:**
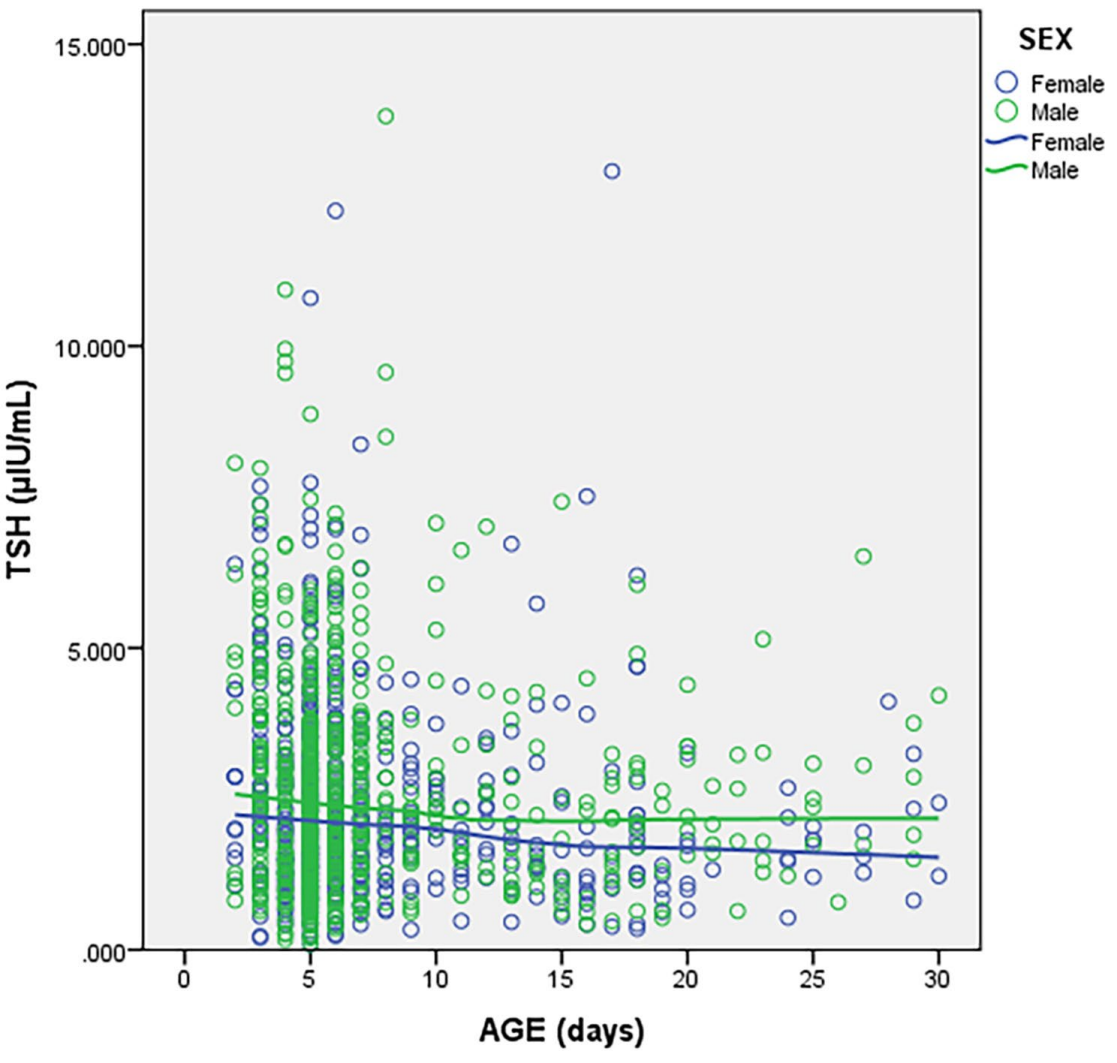
Change in TSH with age

**Fig. 2:**
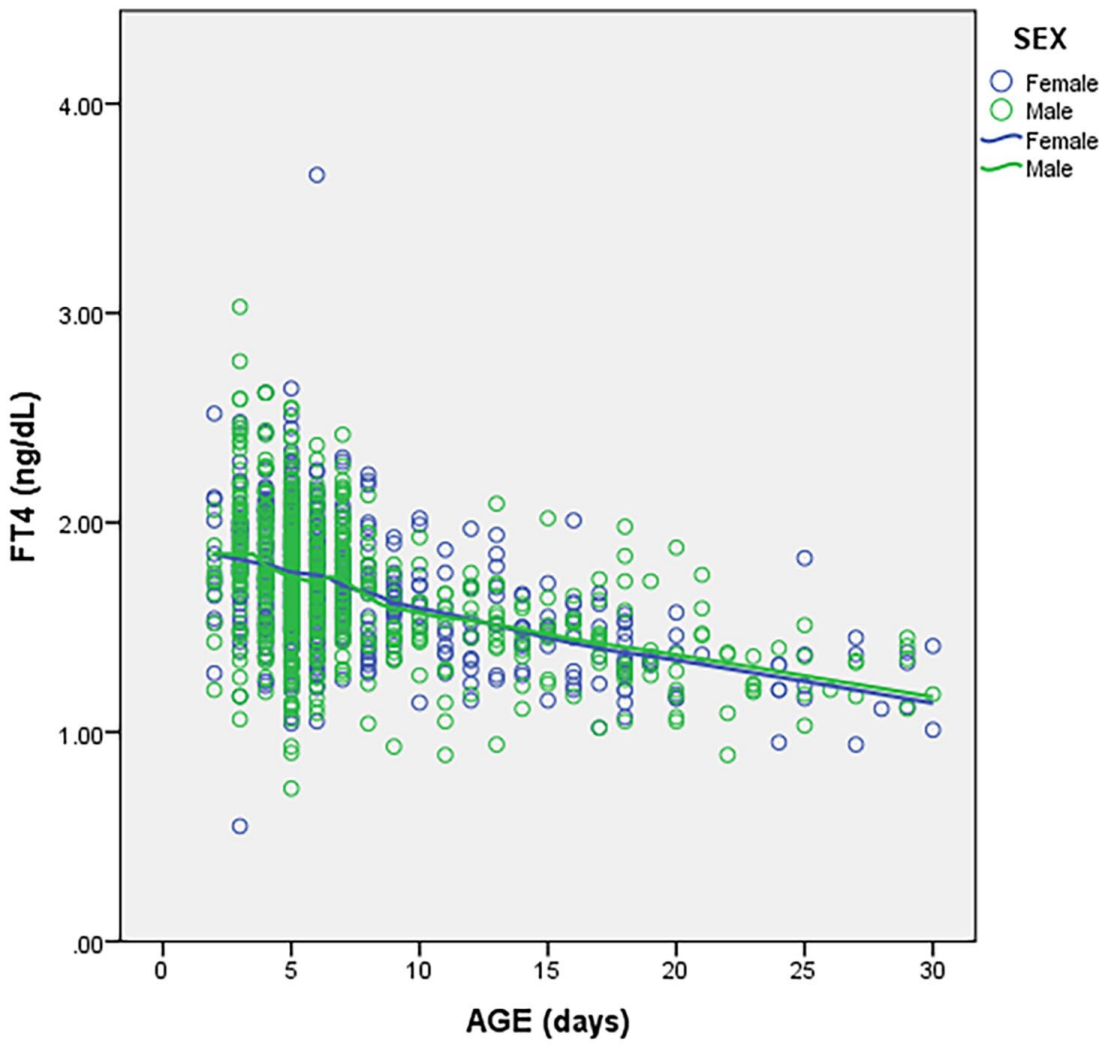
Change in FT4 with age

Both TSH and FT4 declined with increase in age as shown in Figs. 1 and 2 with a more marked decline seen in the first 7 days of life. Inter-individual variation in TSH and FT4 also reduced with increasing age.

The difference in FT4 (p = 0.011) and TSH (p = 0.030) medians between neonates aged 2–4 and 5–7 days was statistically significant. There was a statistically significant difference in FT4 (p < 0.001) and TSH (p = 0.022) medians between neonates aged 2–7 days and 8–30 days. Comparison of medians between males and females revealed a statistically significant difference for TSH in the age group 8–30 days (p = 0.032) with females having lower values. There were no sex differences for FT4 across the various age stratifications. The derived RIs for FT4 and TSH are shown in Table 2.

**Table 2:** Age and sex specific serum neonatal reference intervals for TSH and FT4

			M + F			M			F		
Item	Units	Age (days)	n	LL	UL	n	LL	UL	n	LL	UL
TSH	µIU/mL	2 to 4	244	**0.403**	**7.942**	138	0.334	9.654	106	0.356	7.154
		5 to 7	660	**0.418**	**6.319**	341	0.428	6.088	319	0.417	6.971
		2 to 7	904	0.418	6.973	479	0.416	7.041	425	0.418	6.973
		8 to 30	314	0.473	7.015	154	**0.609**	**7.557**	160	**0.420**	**6.189**
FT4	ng/dL	2 to 4	244	**1.19**	**2.59**	138	1.18	2.62	106	1.20	2.49
		5 to 7	660	**1.21**	**2.29**	341	1.13	2.30	319	1.23	2.29
		2 to 7	904	1.20	2.42	479	1.16	2.43	425	1.23	2.32
		8 to 30	314	**1.02**	**2.01**	154	0.94	1.99	160	1.02	2.02

Recommended RIs are in bold

**Fig. 3:**
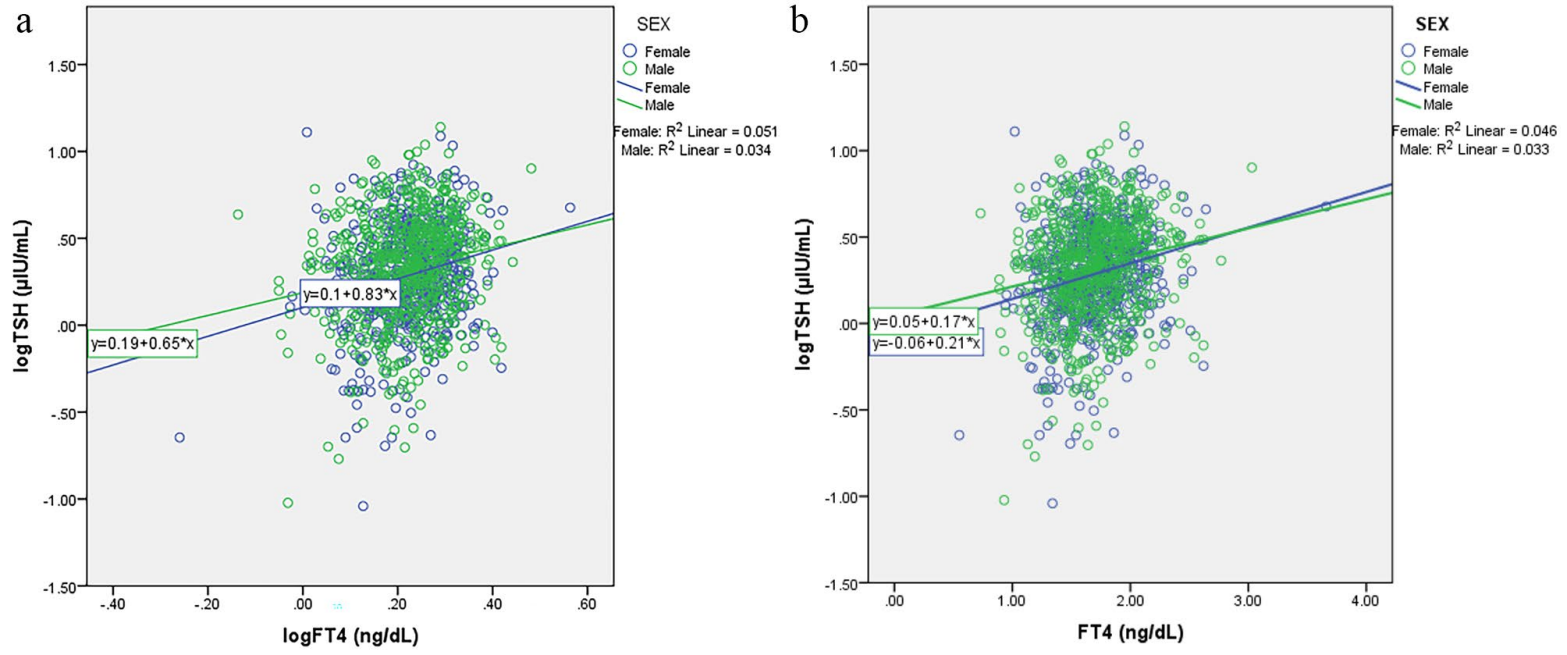
**a** Linear regression plot of logTSH and logFT4, **b** Linear regression plot of logTSH and FT4

There was a weak positive correlation between logTSH and logFT4 which was statistically significant (r_s_ (1216) = 0.189, p = < 0.001) and between logTSH and FT4 (r_s_ (1216) = 0.189, p = < 0.001). An increase in FT4 or logFT4 was associated with an increase in logTSH as shown in Fig. 3. The positive correlation persisted even after stratifying by age and sex.

## Discussion

We determined RIs for TSH and FT4 using hospital derived data from neonates undergoing routine screening for CH at AKUHN. Our RIs differ from those published by the Canadian CALIPER study as we have demonstrated age and sex differences within the neonatal period. The CALIPER study published a single RI from birth to 18 years of age for TSH and FT4 done on the Atellica® IM analyzer as they found no statistically significant differences across age and sex stratifications [7]. This is despite previously publishing age specific TFT RIs for infants, children and adolescents based on tests done on analyzers from other manufacturers [12, 13]. This finding is not in keeping with what has been observed in other studies especially for TSH which changes significantly with increase in age in the neonatal period [4, 8, 14]. The CALIPER TSH upper limit of 4.409 µIU/mL would result in 145 (11.9%) neonates in the current study being classified as having elevated TSH compared to 29 (2.4%) using our sex and age specific RIs. We derived our RIs for both TSH and FT4 from 1218 neonates compared to the CALIPER study which published FT4 and TSH RIs from 810 and 828 children from birth to 18 years respectively but didn’t indicate how many neonates were included. Given the number of neonates included in the current study, we are better placed to explore age and sex as sources of variation and as such our RIs are probably more representative of the physiology of TSH and FT4 in the neonatal period.

We have previously published neonatal RIs for FT4 and TSH determined using a Roche cobas6000® analyzer and demonstrated statistically significant sex differences in TSH RIs for neonates aged 0-14days with RIs of 0.59–12.84 and 0.56-11.00 µIU/mL for males and females respectively [4]. In the current study, female neonates also had lower TSH values in the age group 8–30 days compared to males. Sex differences were however not evident for FT4. A similar phenomenon has been described in Australia by Hadlow et al. where for FT4 within the reference range, median TSH was higher in males than in females though children below 1 year of age were excluded in this study [15]. Both TSH and FT4 in the current study progressively decreased with increasing age with the decline being more rapid in the first week of life which is in keeping with what has previously been described [8]. We subsequently stratified the RIs into three groups (2–4, 5–7 and 8–30 days) to reflect this observation as the difference in TSH and FT4 medians between these age groups was statistically significant with lower values found in the 8–30 days age group. Zurakowski et al. carried out a study in children in the US and reported a more rapid decline in TSH and tri-iodothyronine (T3) among females, a difference that was statistically significant. No sex difference was observed in the rate of decline in FT4 [10]. Indeed, age and sex are key sources of variation for TSH and FT4 in neonates and this should be taken into consideration when providing RIs in order to ensure correct interpretation.

There was a positive correlation between logTSH and FT4 which was unexpected given that the relationship between TSH and FT4 has previously been described as inverse log linear with a 2-fold change in FT4 producing a 100-fold change in TSH in the opposite direction [16, 17]. Hadlow et al. carried out a large cross-sectional study and found that the relationship between logTSH and FT4 was best described as complex non-linear defined by two negative sigmoid curves [15]. Other studies have also defined the relationship as being non-linear or complex but one cannot extrapolate their findings to a neonatal population as they only included adults [18, 19]. Taylor et al. conducted a longitudinal analysis of a large population birth cohort in the United Kingdom and demonstrated substantial changes in the pituitary–thyroid axis over childhood. Specifically, there was a positive relationship between TSH and free tri-iodothyronine (FT3) and inverse relationship between TSH and FT4 at 7 and 15 years of age even after adjusting for possible confounders [20]. They hypothesized that the increase in FT3 might be related to factors not related to the pituitary-thyroid axis including endocrine, pre-pubertal or body mass. It is possible that the relationship between TSH and FT4 in the neonatal period is complex given the dynamic nature of thyroid physiology due to changes in the sensitivity of thyrotropin releasing hormone neurons and thyrotropes to thyroid hormone feedback before a nadir is reached. Assaying FT3 would have enabled us to determine how the positive correlation between TSH and FT4 impacts on the kinetics of FT3 in the neonatal period.

The use of indirect methods in deriving RIs is ideal for the paediatric age group given the inherent challenges in collecting suitable samples from neonates as well as sensitivities around recruitment of neonates to research studies. Hospital based data collected as part of routine screening can go a long way in generating RIs that can inform clinical practice [21]. Use of existing data to derive RIs is quick and cost effective compared to using direct methods where reference individuals are recruited prospectively based on pre-defined inclusion criteria. However, using data from hospital databases to derive RIs has an inherent challenge of distinguishing the healthy reference individuals from those with overt or sub-clinical disease, and whether the presence of diseased individuals influences the RIs [22]. The use of indirect methods where the data source is individuals undergoing routine screening needs to take into consideration the prevalence of the condition being screened for. Unfortunately, screening for CH is not well established in SSA due to other more pressing healthcare needs and this has contributed to the paucity of data on RIs for TFTs as well as on the incidence of CH in Africa. At AKUHN, we are fortunate to have a screening programme for CH which affords us the opportunity to derive RIs for TFTs. Given the retrospective nature of this study, invariably a few neonates with overt or sub-clinical illnesses that could alter TSH and FT4 values may have been included as part of the reference population. However, based on our previous experience, the percentage of such children among those attending a well-baby clinic is very low and would not significantly alter the derived RIs. In order to reduce the possibility of including neonates who may have had thyroid dysfunction we only included the most recent TSH and FT4 results for those with more than one set of results in the neonatal period as this would most likely reflect the ‘normal’ values for those on follow up for possibly deranged initial results.

This study has several limitations that need to be considered when adopting the findings. The study was retrospective and we didn’t have any follow up data to determine whether any neonates were eventually diagnosed with a thyroid disorder. We also didn’t review individual medical records hence cannot guarantee that all neonates were term healthy babies. However, neonates attending the well-baby clinic are generally healthy and are on follow up primarily to monitor growth, attainment of milestones and to receive vaccinations. Thyroid function testing is discouraged in neonates who have an acute illness hence it is unlikely that the study dataset includes results from sick neonates. There is a possibility that a few of the neonates may have been pre-term but this proportion would be low as routine screening for CH in pre-terms is only performed on attainment of an age equivalent to a term baby. Screening for CH at AKUHN is also not compulsory hence those who opt in may not necessarily be reflective of the general population. Nevertheless, we derived RIs from 1218 children with paired FT4 and TSH results which is adequate to derive population specific RIs considering that CLSI recommends a minimum of 120 reference individuals per stratification. Another limitation is that both FT4 and TSH continuously decline in the neonatal period and therefore any proposed age stratification will have an element of subjectivity. We stratified our dataset into 3 age bins based on the observed trend of a more rapid decline for both FT4 and TSH within the first week of life compared to the subsequent weeks which is in keeping with what has previously been described [4, 8]. Indeed, the median difference for both FT4 and TSH between the age stratifications chosen was statistically significant. The other limitation is that our RIs are only relevant for laboratories that are performing these tests on a Siemens Atellica® IM analyzer and should not be used to interpret FT4 and TSH values obtained from other analyzers.

Our study fills an important gap as it is, to the best of our knowledge, the only study from Africa that has proposed neonatal TSH and FT4 RIs for the Siemens Atellica® IM analyzer. Unlike the CALIPER study that reported no significant age and sex differences in RIs for TSH and FT4 from birth to 18 years [7], we found age and sex differences in RIs for TSH and age differences for FT4 in the neonatal period.

## Conclusion

Our neonatal RIs for TSH and FT4 are different to those published or recommended by Siemens. This emphasizes the need for population specific RIs or at the very least verification of what is available prior to adoption. The RIs will serve as a guide for the interpretation of TFTs in neonates from sub-Saharan Africa where routine screening for congenital hypothyroidism using serum samples is done on the Siemens Atellica® IM analyzer.

## Data Availability

The datasets used and/or analysed during the current study are not publicly available as it contains sensitive patient data but is available from the corresponding author on reasonable request.

## Abbreviations

AKUHN: Aga Khan University Hospital, Nairobi
CH: Congenital hypothyroidism
FITC: Fluorescein isothiocyanate
FT3: Free tri-iodo thyronine
FT4: Free thyroxine
IQR: Interquartile range
RI: Reference interval
T3: Tri-iodo thyronine
T4: Thyroxine
TFT: Thyroid function test
TSH: Thyroid stimulating hormone
